# Supplementary material for: Patterns of Intron Gain and Loss in Fungi
Source: PLoS Biol. 2004 Nov 30;2(12):e422. doi: 10.1371/journal.pbio.0020422 (PMC532390; doi:10.1371/journal.pbio.0020422)
Supplement: Table S1 — Also available at http://genes.mit.edu/NielsenEtAl/. (4.3 MB ZIP). [file pbio.0020422.st001.zip › NielsenEtAl/html/910.html]

AN5486.1.NCU06726.1.MG06196.1.FG10250.1


```
 CLUSTAL W (1.82) Multiple Sequence Alignments - Introns Inserted


Sequence 1: NCU06726.1	306 aa
Sequence 2: FG10250.1	301 aa
Sequence 3: MG06196.1	307 aa
Sequence 4: AN5486.1	422 aa
Alignment Length: 436 aa
Number Identitical Residues: 166 aa
Alignment Score (without introns) 7888


MG06196.1 	MASSAEKTHEPVLKQPVKLACIQLASG~ADKAANLSHARDKVIEASRAGAKIILLPECFN
NCU06726.1	MAS--STKHPILLKKPVKLACIQLASG~ADKSANLSHAADKVREAASGGANIVVLPECFN
FG10250.1 	MSS--ATAP--VLKQRVKLSLIQLASG~SDKKANLDSAASHVARVASSGAKIVVLPECFN
AN5486.1  	MTA--------LLKKPLKLALVQLASG1ADKAANLAHARTKVLEAAKAGASLIVLPECFN
          	*::        :**: :**: :***** :** ***  *  :* ..: .**.:::******

MG06196.1 	SPYGCDYFPSYAEALLPSPPTRGQSPSYHALSAMAKETGAYLIAGSIPELVDGASGDDKD
NCU06726.1	SPYGCDFFPSYAEQLLPSPPTVEQSPSFHALSAMARDNGIYLVGGSIPELAIEEGTEDK-
FG10250.1 	SPYGTDHFPQYAETLLPSPPSKDDAPSYHALSSMAVDNKVYLIGGSIPEY-----SPDT-
AN5486.1  	SPYGTQYFPKYAETLLPSPPTKEQSPSYHALSALAAEAKAYLVGGSIPEL------ETTS
          	**** :.**.*** ******:  ::**:****::* :   **:.*****         ..

MG06196.1 	KKYYNTALVFSPSGDLLATHRKVHLFDIDIPGKITFRESDVLSPGNEVTIVDLPPYGKVG
NCU06726.1	KTYYNTSLVFGPDGKLLASHRKVHLFDIDIPGKIKFKESDVLSPGNSVTLVDLPDYGRIA
FG10250.1 	KKYYNTTLIFGPDGSLLGTHRKVHLFDIDIPGKITFRESDILSPGNKVTLVDLPEYGKIA
AN5486.1  	KKYYNTSLVFSPSGALIGTHRKTHLFDIDIPGKITFKESEVLSPGNQLTIVDLPDYGKIG
          	*.****:*:*.*.* *:.:***.***********.*:**::*****.:*:**** **::.

MG06196.1 	VAICYDIRFPELATIAARKGAFALLYPGAFNMTTGPLHWELLARARAVDNQLYVALCSPA
NCU06726.1	VAICYDIRFPELAMIAARKGCFALVYPGAFNTTTGPLHWRLQGQARAMDNQIYVALCSPA
FG10250.1 	VAICYDVRFPELATIAARKGAFALIYPGAFNTTTGPLHWQLLGRARAADNQLYVALCSPA
AN5486.1  	LAICYDIRFPEPGMTAARKGAFALIYPGAFNTTTGPLHWQLLARARAVDNQVYVALCSPA
          	:*****:**** .  *****.***:****** *******.* .:*** ***:********

MG06196.1 	RDLKASYHAWGHSLVADPMAEVLSQADDVETIVYADLDPEAIEGPRTRIPLRDQRRFDVY
NCU06726.1	RDISASYHAYGHSLIVDPMARVLVEAEESETIVSAELDGTKIEEARSGIPLRDQRRFDIY
FG10250.1 	RAETG-YPAYGHSLVADPMAQVQVEADEKETTVDWELDPEKITEARKAIPLNTQRRFDVY
AN5486.1  	RDTTATYHAYGHSLVADPSAKILSEAEESETIVYADLDPETIESTRKGIPIYTQRRFDLY
          	*  ..:* *:****:.** *.:  :*:: ** *  :**   *  .*. **:  *****:*

MG06196.1 	PD~IS----QPG~A---~-----------------------------~------------
NCU06726.1	PD~VS----QAK0PFF-~-----------------------------~------------
FG10250.1 	PD~VS----EGK~VQFE~ES---------------------------~------------
AN5486.1  	PD0STTSANHSR~TPFN1MSNVQTGKKQRSAIADVVTREYTINLHKR0THGVSFKKRAPR
          	**  ::::..      .  :. .:.... :: :.  : . : .  .  : . : .. :. 

MG06196.1 	-------------~----------------------------------------------
NCU06726.1	-------------~----------------------------------------------
FG10250.1 	-------------~----------------------------------------------
AN5486.1  	AIKEIRAFATRAM0GTTDVRLDPQLNKKVWEAGIKGVPFRLRVRISRKRNDEEGAKEKLY
          	: ..  : :: :  .::.   ... ...  .:. .. .       : . .....:...  

MG06196.1 	----------------------
NCU06726.1	----------------------
FG10250.1 	----------------------
AN5486.1  	SYVQAVNVKEPKGLQTTVVDEE
          	:  .: . ..... .::  ...
```
